# Comparative Study Between Fosaprepitant and Palonosetron in the Prophylaxis of Postoperative Nausea and Vomiting in Women Undergoing Laparoscopic Cholecystectomy: Prospective, Randomized and Double-Blind Study

**DOI:** 10.3389/fphar.2022.915347

**Published:** 2022-05-11

**Authors:** Estêvão Luiz Carvalho Braga, Nubia Verçosa, Ismar Lima Cavalcanti

**Affiliations:** ^1^ Department of General and Specialized Surgery, Medical Sciences Postgraduate Program, Fluminense Federal University, Niterói, Brazil; ^2^ Department of Surgery/Anaesthesiology, Surgical Sciences Postgraduate Program, Federal University of Rio de Janeiro, Rio de Janeiro, Brazil

**Keywords:** nausea, vomiting, postoperative nausea and vomiting, fosaprepitant, palonosetron

## Abstract

**Objective:** To test the hypothesis that the single use of fosaprepitant is not inferior to the use of palonosetron as antiemetic prophylaxis in the first 48 h after surgery in women undergoing laparoscopic cholecystectomy.

**Method:** Eighty-eight nonsmoking women (American Society of Anesthesiologists physical status I or II) aged between 18 and 60 years who underwent laparoscopic cholecystectomy received 150 mg of fosaprepitant or 75 μg of palonosetron, administered intravenously after the induction of general anesthesia.

**Results:** In the fosaprepitant group and in the palonosetron group, 13.6 and 18.2% of the patients, respectively, vomited in the first 48 h after surgery (*p* = 0.560). There were no differences between groups in the total frequency and intensity of nausea, number of complete responders, need for rescue medication, time required for the first rescue medication dose or number of adverse events.

**Conclusion:** The administration of a single dose of fosaprepitant after the induction of anesthesia was as effective as the administration of a single dose of palonosetron for the prophylaxis of vomiting in the first 48 h after surgery in women undergoing laparoscopic cholecystectomy.

**Clinical Trial Registration**: (https://www.clinicaltrials.gov/ct2/show/NCT03586817), identifier (NCT03586817).

## Introduction

Despite continuous research and the development of new drugs and techniques, postoperative nausea and vomiting (PONV) are frequent, cause unexpected hospitalizations, delay hospital discharge, increase hospital costs and generate patient dissatisfaction (Horn et al., 2014; Kranke et al., 2020). Laparoscopic surgery is cited in the literature as a risk factor for PONV, with an incidence of up to 75% in cases where prophylactic drugs are not used (Grover et al., 2009; Bala et al., 2014).

Aprepitant is an NK-1 receptor inhibitor that is capable of blocking the action of substance P at its action sites. Aprepitant has a lifespan of up to 40 h but has the disadvantage of being available in commercial version only in tablets, a factor that limits its use in anesthesiology. Fosaprepitant is a pro-drug with a half-life of up to 13 h, which allows its use as a single dose. When administered by the intravenous route (iv), it is rapidly converted by the effect of the first hepatic passage into aprepitant (Gan et al., 2014; Gan et al., 2020).

Palonosetron is a second-generation 5-HT3 antagonist, initially used in the prevention of nausea and vomiting associated with chemotherapy (Affronti and Bubalo, 2014; Nasir and Schwartzberg, 2016). It differs from other antagonists due to its allosteric property and high affinity for serotonergic receptors. Its high plasma half-life (40 h) allows the administration of a single dose in the perioperative period, which may make the therapy more effective and cost-effective when compared to other drugs in its group (Rojas et al., 2014; Gouveia de Araujo Ferreira et al., 2020).

Studies have shown that both drugs are more effective than ondansetron for the prophylaxis of nausea and vomiting after chemotherapy and in PONV (Gan et al., 2007; Rojas et al., 2008; Alonso-Damián and Anguiano-García, 2012). Based on a literature search, this is the first study that compares fosaprepitant and palonosetron for the prevention of PONV, making this study unprecedented.

The primary objective of this study was to evaluate the frequency of vomiting in the first 48 h postoperatively after the administration of 150 mg of fosaprepitant or 75 µg of palonosetron in women undergoing videolaparoscopic cholecystectomy. The secondary objectives were to evaluate the frequency of vomiting at other predefined times; evaluate the frequency of nausea in the first 48 h after surgery and at other predefined times; quantify the intensity of nausea; quantify the number of complete responders; evaluate the need for rescue medication; measure the time required for the administration of the first rescue dose; and quantify the adverse effects sleepiness, headache, dizziness and weakness.

## Materials and Methods

This prospective, randomized and double-blind clinical trial was conducted after approval by the Research Ethics Committee of Bonsucesso Federal Hospital, Rio de Janeiro, under number 48149215.0.0000.5253, opinion no. 2,734,633, on 25 June 2018 and registered at Clinicaltrials.gov (NCT03586817) on 2 March 2019.

Ninety women aged between 18 and 60 years, nonsmokers, physical status classified by the American Society of Anesthesiologists (ASA) scale as I or II, and Apfel score ≥ 2, undergoing elective laparoscopic cholecystectomy were recruited and distributed into two groups: Group A received 150 mg) of fosaprepitant, and Group B received 75 µg) of palonosetron. The following exclusion criteria were adopted: participation in another study in the last month, body mass index (BMI) > 35 kg/m^2^, occurrence of episodes of nausea or vomiting within 24 h prior to surgery, motion sickness, previous PONV, people who smoke, people with alcoholism, use of corticosteroids, psychoactive or antiemetic drugs, hypersensitivity to the study medications, serious kidney, liver, lung, heart, brain or bone marrow disease, and conversion from laparoscopic cholecystectomy to conventional cholecystectomy.

An informed consent form was presented and signed, on an outpatient basis, by each of the volunteer participants, who were instructed about the risks and benefits of participating in this study. The patients were randomized into two groups using GraphPad Prism Quickcalcs (GraphPad Software®, Inc., La Jolla, CA, United States). The randomization was kept in a sealed brown envelope, and only one nurse not participating in the study had access to the envelope and prepared the antiemetic solutions, in a dilution of 250 ml of saline solution, that were administered by the iv route in a single dose after anesthetic induction.

The patients were preoxygenated with 100% oxygen for 5 min, and anesthetic induction was performed by the iv administration of fentanyl (3 μg kg^−1^), lidocaine (1.5 mg kg^−1^) and propofol (2 mg kg^−1^). Tracheal intubation was performed 3 min after the 4 administration of 0.6 mg kg^−1^ rocuronium. The patients received 150 mg of fosaprepitant or 75 μg of palonosetron, administered intravenously after the induction of general anesthesia.

Anesthesia was maintained with sevoflurane and oxygen/air (50%) at 2 L/min, with an inspired sevoflurane concentration of approximately 2.0%. Remifentanil (0.05 μg kg^−1^ min to 0.3 μg kg^−1^ min) was administered intraoperatively when the heart rate or blood pressure of a patient increased above 20% of baseline values. Additional 4 doses of rocuronium were also administered based on need and clinical criteria.

Patients received parecoxib (40 mg), dipyrone (50 mg kg^−1^), ketamine (0.3 mg kg^−1^) and clonidine (1 μg kg^−1^) after tracheal intubation. Pneumoperitoneum insufflation with carbon dioxide was limited to an abdominal pressure of 12 mmHg. Before suturing, the surgical wound was infiltrated with 20 ml of 0.5% ropivacaine, and morphine (0.03 mg kg^−1^) was administered). Neuromuscular blockade was reversed with neostigmine (0.04 mg kg^−1^) and atropine (0.02 mg kg^−1^), and the trachea was extubated. The duration of anesthesia, surgery, and pneumoperitoneum insufflation and the volume of Ringer’s lactate used were recorded.

The patients received a clinical visit by the research team at 2, 6, 24 and 48 h after the end of surgery and were asked about the frequency and intensity of nausea, frequency of vomiting, and occurrence of adverse effects (weakness, headache, dizziness and sleepiness). For the purpose of the study, nausea was defined as the unpleasant and involuntary sensation of vomiting, without expulsion of gastric contents, and vomiting was defined as the expulsion of stomach contents. The occurrence of retching (spasmodic and involuntary contractions of the respiratory muscles without the expulsion of gastric contents) was considered vomiting. The intensity of the nausea episodes was measured using a qualitative grading scale that ranged from mild to intense.

In patients from both groups, metoclopramide (10 mg) was used as rescue medication for PONV. Rescue was performed in case of severe nausea and vomiting or at the request of the patient.

A visual analogue scale (VAS) was used to measure pain intensity. The scale ranged from 0 to 10 (where 0 = no pain and 10 = worst possible pain). Morphine (0.03 mg kg^−1^) was administered to patients with pain >4 (moderate pain) based on the VAS.

During the last clinical visit (48 h postoperatively), the researchers analyzed the medical records and collected data on the use of rescue opioids and rescue antiemetics.

### Statistical Analysis

This study is a longitudinal clinical trial with the primary objective of determining whether the incidence of vomiting in the first 48 h after surgery is significantly different between two groups administered antiemetic prophylaxis: fosaprepitant (Group A) and palonosetron (Group B). To calculate the sample size, parameters previously defined by the researchers from knowledge acquired through a pilot study of 33 cases (16 from Group A and 17 from Group B) were necessary. Under the hypothesis of noninferiority of Group A compared to standard Group B, we considered the following parameters: vomiting rate (θ) of Group A of 6.3% and of Group B of 23.5%, significance of 5% (two-tailed), 80% power and maximum acceptable error for equivalence (δ) of 5%. The following hypothesis was tested: H_0_—θA — θB ≥ δ versus H_a_—θA — θB < δ. According to Blackwelder (1982), the minimum number of patients would be 42 for each group for a power of 90%. Considering the probability of loss to follow-up, a total of 90 patients were randomized (45 per group).

The descriptive analysis of the observed data is presented in the form of tables, with the results expressed as the mean, standard deviation, median and interquartile range (Q1 — Q3) for numerical data and as the frequency and percentage for categorical data. The inferential analysis involving the comparison between the two treatment groups consisted of Student’s t test for independent samples, the Mann–Whitney test for numerical data and the chi-square (χ^2^) or Fisher’s exact test for categorical data. The nonparametric method was applied because the surgical data did not have a normal distribution (Gaussian) due to the rejection of the normality hypothesis (Shapiro–Wilk test). The significance level adopted was 5%. Statistical analyses were performed using the statistical software SAS^®^ System, version 6.11 (SAS Institute, Inc., Cary, North Carolina). Regarding the analysis of the 0–24 h and 0–48 h periods, individuals who had more than one episode of nausea or vomiting in the predefined intervals were counted only once.

## Results

**FIGURE 1:**
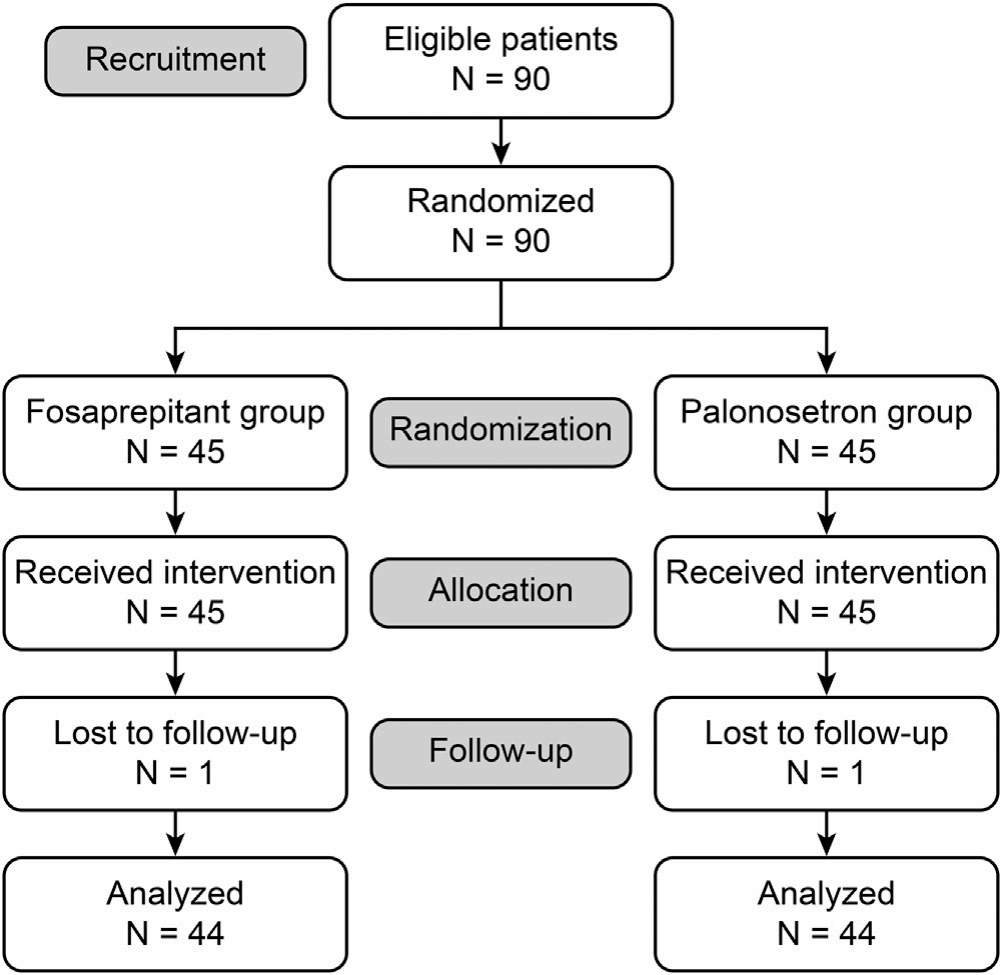
Consort flow-chart.

A total of 90 eligible patients were selected. During the study, there was loss to follow-up in each group, thus resulting in 88 analyzed patients (Figure 1).

There were no differences between the groups regarding the clinical and surgical variables, such as ASA physical status classification, Apfel score, age, weight, height, BMI, anesthesia duration, surgery duration, pneumoperitoneum insufflation duration, total doses of fentanyl, remifentanil and morphine and administered volume of crystalloids (Table 1).

**TABLE 1:** Clinical and surgical variables for the treatment groups.

Variable	Fosaprepitant (*n* = 44)	Palonosetron (*n* = 44)	*p* value
ASA class, *n* (%)			
I	33 (75.0%)	30 (68.2%)	0.478
II	11 (25.0%)	14 (31.8%)	
Apfel score, n (%)			
1	41 (93.1%)	39 (88.6%)	0.458
2	3 (6.9%)	5 (11.4%)	
Age (years), mean ± SD	41.7 ± 10.2	39.5 ± 9.9	0.315
Weight (kg), mean ± SD	72.9 ± 12.8	70.8 ± 12.4	0.434
Height (cm) , mean ± SD	163.5 ± 7.2	162.9 ± 6.8	0.716
BMI (kg/m^2^), mean ± SD	27.2 ± 4.4	26.5 ± 3.7	0.418
Anesthesia duration (min), median (IQR)	115 (90–130)	110 (90–140)	0.903
Surgery duration (min), median (IQR)	83 (74–100)	90 (70–105)	0.538
Insufflation duration (min), median (IQR)	60 (42–77)	60 (45.8–75)	0.738
Total dose of fentanyl (mcg), median (IQR)	225 (200–250)	250 (200–250)	0.344
Total dose of remifentanil (mcg), median (IQR)	0 (0–350)	250 (0–400)	0.211
Total dose of morphine (mg), median (IQR)	2 (2–2)	2 (2–2)	0.800
Volume administered (ml), median (IQR)	1200 (1000–1500)	1200 (1000–1375)	0.440

IRQ, interquartile range; SD, standard deviation. Chi-square, Student’s *t* test or Mann–Whitney test.

There was no statistical difference between the groups studied in the primary outcome frequency of vomiting in the first 48 h postoperatively (*p* = 0.560). The occurrence of vomiting was lower in the fosaprepitant group at 6 and 24 h postoperatively: 0 versus 11.4% (*p* = 0.028). There was no difference, at the 5% level, in the other postoperative variables between the two treatment groups (Table 2) or in the final evaluation variables between the two treatment groups (Table 3).

**TABLE 2:** Nausea and vomiting during the four evaluated periods.

Variable	Fosaprepitant (*n* = 44)		Palonosetron (*n* = 44)		*p* value
	*n*	%	*n*	%	
Period from 0 to 2 h					
Nausea	12	27.3	11	25.5	0.808
Nausea intensity					
Absent	32	72.7	33	75.0	0.574
Mild	8	18.2	5	11.4	
Intense	4	9.1	6	13.6	
Vomiting	0	0	2	4.5	0.247
Period from 2 to 6 h					
Nausea	21	47.7	14	31.8	0.127
Nausea intensity					
Absent	23	52.3	30	68.2	0.267
Mild	14	31.8	8	18.2	
Intense	7	15.9	6	13.6	
Vomiting	5	11.4	3	6.8	0.357
Period from 6 to 24 h					
Nausea	13	29.5	16	36.4	0.496
Nausea intensity					
Absent	31	70.5	28	63.6	0.776
Mild	7	15.9	8	18.2	
Intense	6	13.6	8	18.2	
Vomiting	0	**0**	5	**11.4**	**0.028**
Period from 24 to 48 h					
Nausea	8	18.2	7	15.9	0.776
Nausea intensity					
Absent	36	81.8	37	84.1	0.949
Mild	6	13.6	5	11.4	
Intense	2	4.5	2	4.5	
Vomiting	1	2.3	1	2.3	0.753

Chi-square or Fisher’s exact test.

**TABLE 3:** Final evaluation variables for the treatment groups.

Variable	Fosaprepitant (*n* = 44)		Palonosetron (*n* = 44)		*p* value
	*n*	%	*n*	%	
Overall period					
Nausea 0–24 h	27	61.4	24	54.5	0.517
Nausea 0–48 h	27	61.4	26	59.1	0.827
Nausea intensity 0–48 h					
Absent	17	38.6	18	40.9	0.971
Mild	10	22.7	10	22.7	
Intense	17	38.6	16	36.4	
Vomiting 0–24 h	5	11.4	8	18.2	0.367
Vomiting 0–48 h	6	13.6	8	18.2	0.560
Final evaluation					
Complete responder	17	38.6	18	40.9	0.827
Rescue medication	17	38.6	18	40.9	0.827
Time to rescue medication*	0 (0–165)		0 (0–143)		0.950
Opioid postop	3	6.8	5	11.4	0.357

* Time to rescue medication is expressed as the median and interquartile range (Q1-Q3) and was compared by the Mann–Whitney test.

Chi-square or Fisher’s exact test.

There was no difference, at the 5% level, in adverse effects up to 48 h postoperatively between the two treatment groups (Table 4); however, there was a trend towards a more frequent occurrence of headache in the palonosetron group than in the fosaprepitant group.

**TABLE 4:** Adverse effects in the first 48 postoperative hours for the treatment groups.

Variable	Fosaprepitant (*n* = 44)		Palonosetron (*n* = 44)		*p* value
	*n*	%	*n*	%	
Headache 0–48 h	5	11.5	11	25.0	0.097
Dizziness 0–48 h	8	18.2	3	6.8	0.107
Sleepiness 0–48 h	6	13.6	4	9.1	0.500
Weakness 0–48 h	3	6.8	2	4.5	0.500

Chi-square or Fisher’s exact test.

## Discussion

Nausea and vomiting are two of the main adverse events that occur in the postoperative period and constitute a distressing experience for patients (Gan et al., 2014; Kranke et al., 2020). They cause significant increases in the length of hospital stay, in postdischarge readmissions and in costs for the health system (Candelario and Lu, 2016; Kranke et al., 2020). The management of PONV is a complex process, and several studies have been conducted focusing on this topic (Gan et al., 2014; Kono et al., 2018).

The emergence of new drugs for the antiemetic management of patients undergoing cycles of highly emetogenic chemotherapy has led to their use in the anesthetic and postoperative context (Candelario and Lu, 2016; Nasir and Schwartzberg, 2016; Kono et al., 2018). To date, there are few studies available in the literature on the use of fosaprepitant in the context of PONV prophylaxis (Kakuta et al., 2015; Soga et al., 2015; Atsuta et al., 2017), and we did not find a specific comparison between fosaprepitant and palonosetron in the main databases.

Regarding the primary outcome of this study, postoperative vomiting in the 0–48 h period, we found a frequency of 13.6% in the fosaprepitant group. This result is similar to the findings by Atsuta et al. (2017), who reported a frequency of 12.8%, also using the opioids fentanyl and remifentanil during anesthesia. However, the study by Atsuta et al. (2017) was performed in the neurosurgery context and included the first 72 h after surgery. In two other studies using fosaprepitant in patients undergoing general anesthesia, Soga et al. (2015) in the 0–72 h period and Kakuta et al. (2015) in the 0–48 h period, the frequency of vomiting was zero. Methodological differences between our study and those by Soga et al. (2015) and Kakuta et al. (2015) may explain these results. We specifically included female patients undergoing laparoscopic cholecystectomy; in contrast, Soga et al. (2015) included patients undergoing gynecologic surgery by an abdominal approach, and Kakuta et al. (2015) included patients of both sex undergoing orthopedic lower limb surgery.

In the present study, no patient in the fosaprepitant group vomited in the first two postoperative hours, and the individuals who vomited in this group were concentrated in the 2–6 h time interval. In the 6–24 h postoperative period, 11.4% of the patients in the palonosetron group vomited, compared to no patients in the fosaprepitant group.

In the present study, in which two antiemetics were investigated as monotherapy, although there was a low frequency of postoperative vomiting, most patients in both groups reported nausea (fosaprepitant 61.4% and palonosetron 59.1%), reinforcing the need for double or triple antiemetic prophylaxis in individuals highly susceptible to PONV (Gan et al., 2014; Gan et al., 2020), thus preventing the discomfort caused by nausea and increasing the degree of patient satisfaction with antiemetic therapy (Gan et al., 2014; Gouveia de Araujo Ferreira et al., 2020). Other studies (Bala et al., 2014; Kakuta et al., 2015; Soga et al., 2015; Carvalho Braga et al., 2019) using antiemetic monotherapy with fosaprepitant or palonosetron also reported a high number of individuals with postoperative nausea. Soga et al. (2015) and Kakuta et al. (2015) reported frequencies of postoperative nausea of 71 and 53%, respectively, after antiemetic prophylaxis with fosaprepitant (150 mg) Carvalho Braga et al. (2019) and Bala et al. (2014) reported postoperative nausea frequencies of 60 and 42.9%, respectively, in patients who underwent laparoscopic cholecystectomy using antiemetic prophylaxis with 75 mcg of palonosetron.

Soga et al. (2015), Kakuta et al. (2015), and Murakami et al. (2017) compared fosaprepitant with ondansetron and did not observe a significant difference between the number of complete responders and the need for rescue medication between the groups studied. Although in our study the comparison was between fosaprepitant and palonosetron, a second-generation serotonergic antagonist, we found similar results.

Moon et al. (2014) performed a direct comparison between monotherapy with palonosetron (75 mcg) and aprepitant (40 mg oral) for antiemetic prophylaxis in the first 48 h after surgery in patients undergoing laparoscopic gynecological surgery, also using metoclopramide as a rescue medication. Methodologically, because that study compared palonosetron with an NK-1 receptor inhibitor and included female patients undergoing laparoscopic surgery, it most resembles ours. As in our study, when comparing the second-generation serotonergic antagonist with the NK-1 inhibitor, there was no difference between the number of complete responders (74 vs. 77%) or the number of patients who required rescue medication (27.7 vs. 28.2%).

Regarding the adverse effects investigated (headache, dizziness, sleepiness and weakness) there were no differences between the groups studied. Twenty-five percent of the patients in the palonosetron group experienced headache. Headache is the most commonly observed adverse effect in patients using serotonergic antagonists (De Leon, 2006).

The present study has some limitations. All participants included were female. The incidence of PONV is higher in women, and these results should not be extrapolated to men (Candelario and Lu, 2016). The total time studied was limited to the first 48 h after surgery. Emetogenic drugs such as opioids and sevoflurane were used during anesthesia. In this study, prophylaxis with antiemetic monotherapy was performed. The main consensus and guidelines for PONV prophylaxis recommend the use of double or triple prophylactic pharmacotherapy in patients at high risk of PONV (Gan et al., 2020; Kranke et al., 2020). Due to the scarcity of studies comparing the drugs in the studied context, new studies comparing them, both in monotherapy and in combination with other antiemetic agents, should be performed.

We conclude that the administration of a single dose of fosaprepitant after the induction of anesthesia was as effective as the administration of a single dose of palonosetron for the prophylaxis of vomiting in the first 48 h postoperatively in women undergoing laparoscopic cholecystectomy.

## Data Availability

The raw data supporting the conclusions of this article will be made available by the authors, without undue reservation.

## Abbreviations

ASA, (American Society of Anesthesiologists); BMI, (body mass index); PONV, (postoperative nausea and vomiting); 5-HT3, (serotonin type 3 receptor); IV, (intravenous); VAS, (visual analogue scale); χ^2^, (chi-square).
